# Alkaline Earth Metal Template (Cross‐)Coupling Reactions with Hybrid Disila‐Crown Ether Analogues

**DOI:** 10.1002/chem.201904209

**Published:** 2019-11-04

**Authors:** Fabian Dankert, Carsten Donsbach, Julia Rienmüller, Roman M. Richter, Carsten von Hänisch

**Affiliations:** ^1^ Fachbereich Chemie and Wissenschaftliches Zentrum für Materialwissenschaften (WZMW) Philipps-Universität Marburg 35032 Marburg Germany

**Keywords:** alkaline earth metals, cross-coupling, crown ethers, ligand design, Si−O bond activation

## Abstract

Alkaline earth metal iodides were used as templates for the synthesis of novel silicon‐based ligands. Siloxane moieties were (cross‐)coupled and ion‐specific, silicon‐rich crown ether analogues were obtained. The reaction of 1,2,7,8‐tetrasila[12]crown‐4 (**I**) and 1,2‐disila[9]crown‐3 (**II**) with MgI_2_ yielded exclusively [Mg(1,2,7,8‐tetrasila[12]crown‐4)I_2_] (**1**). The larger Ca^2+^ ion was then employed for cross‐coupling of **I** and **II** and yielded the complex [Ca(1,2,7,8‐tetrasila[15]crown‐5)I_2_] (**2**). Cross‐coupling of **I** and 1,2,4,5‐tetrasila[9]crown‐3 (**III**) with SrI_2_ enables the synthesis of the silicon‐dominant 1,2,4,5,10,11‐hexasila[15]crown‐5 ether complex of SrI_2_ (**3**). Further, the compounds [Sr(1,2,10,11‐tetrasila[18]crown‐6)I_2_] (**4**), [Sr(1,2,13,14‐tetrasila[24]crown‐8)I_2_] (**5**), and [Sr(1,2,13,14‐tetrasila‐dibenzo[24]crown‐8)I_2_] (**6**) were obtained by coupling **I**, 1,2‐disila[12]crown‐4 (**IV**) or 1,2‐disila‐benzo[12]crown‐4 (**V**), respectively. Using various anions, the (cross‐)coupled ligands were also observed in an X‐ray structure within the mentioned complexes. These template‐assisted (cross‐)couplings of various ligands are the first of their kind and a novel method to obtain macrocycles and/or their metal complexes to be established. Further, the Si−O bond activations presented herein might be of importance for silane or even organic functionalization.

## Graphical abstract

**Templated synthesis**: Alkaline earth metal iodides were used as templates for the synthesis of novel silicon‐based ligands. Siloxane moieties were (cross‐)coupled and ion specific, silicon‐rich crown ether analogues were obtained. The coupling of various ligands gives an outlook on Group 2 ion‐catalyzed macrocycle and/or silane syntheses.

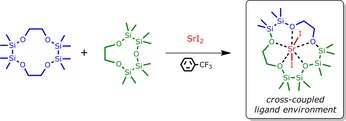

## Introduction

It is well known for crown ethers to form coordination compounds with metal centers across a wide range of the periodic table. These complexes are generally very stable and these polyethers as well as related systems such as cryptands, or in general, multidentate ligands, gained many fields of applications since their discovery in the mid‐sixties. The synthesis of such polyethers, however, is often not trivial because the molecules of the starting materials need to be brought into a suitable conformation for the formation of a specific (most often cyclic) product. The synthesis of these cyclic ethers is therefore mostly metal‐template assisted.^1^

**Scheme 1:**
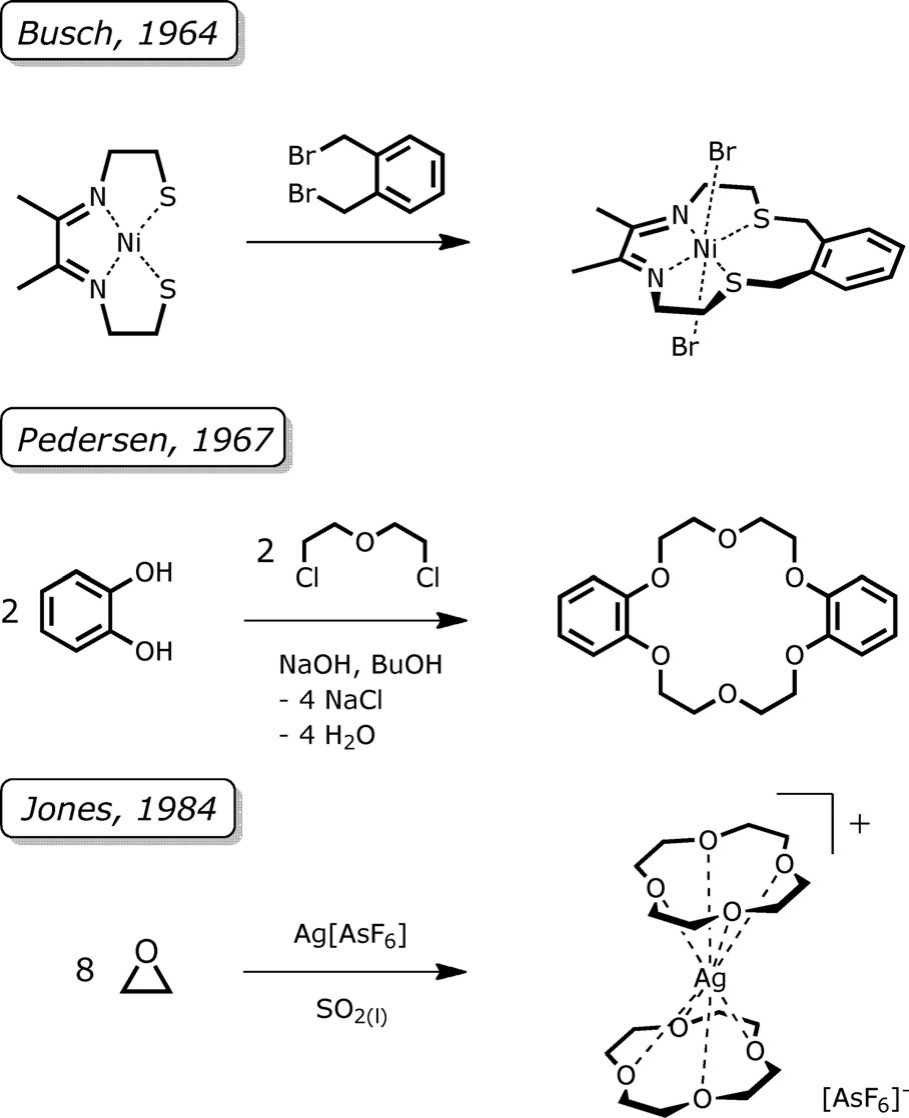
A selection of metal‐template syntheses of cyclic organic crown‐type ligands. In all examples, the metal center acts as a convex template to promote ring closure and/or expansion.^2^, ^3^, ^4^

**Scheme 2:**
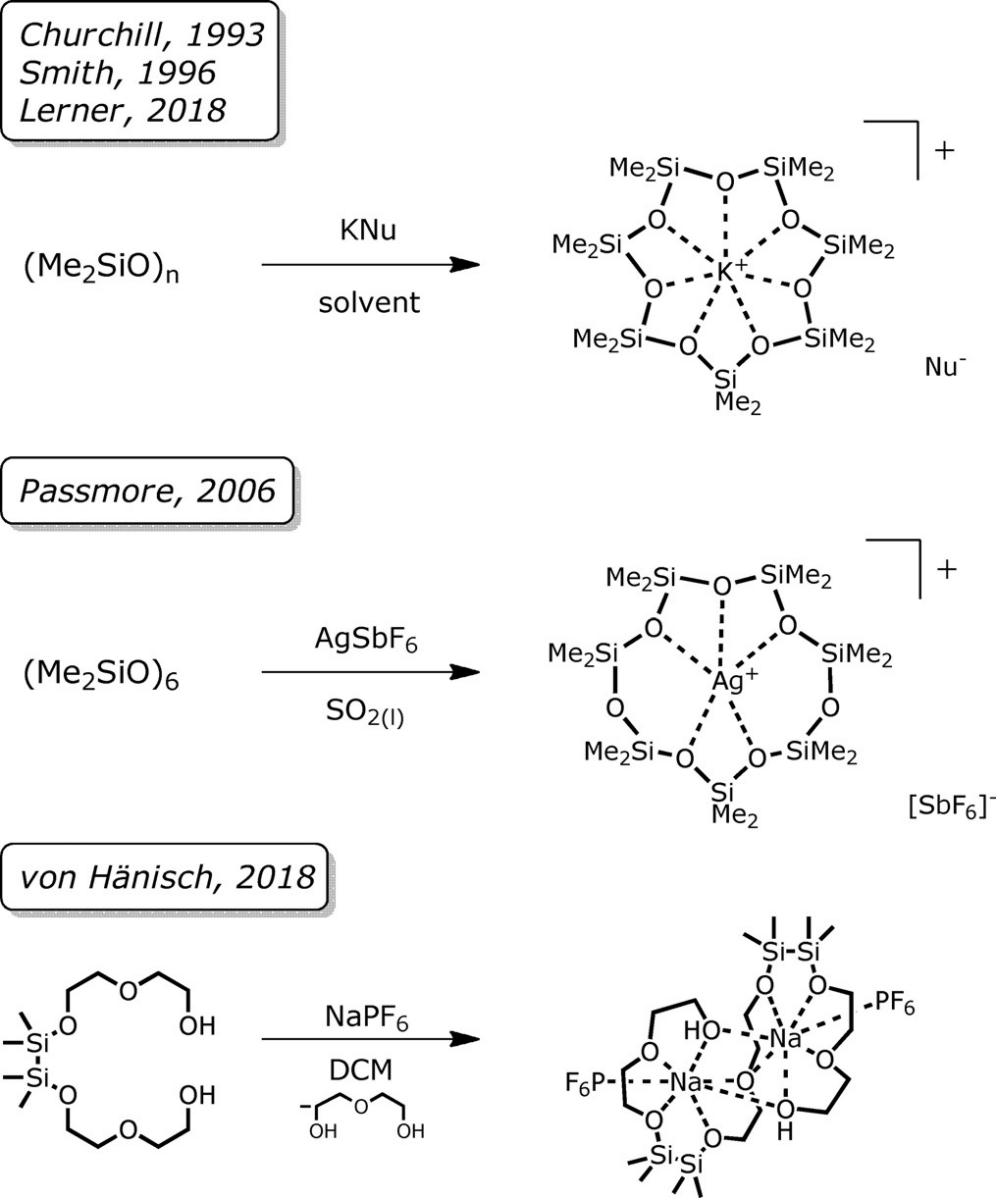
A selection of metal‐template syntheses of cyclic sila‐crown‐type ligands. In all examples, the metal center acts as a convex template to promote ring expansion. For details, please see the cited literature.^6^, ^7^, ^11^, ^12^, ^13^

Templates are known to provide a suitable coordination sphere for the starting materials, which favors the linkage to ring closure and allows obtaining cyclic ligand systems in much higher yields.^1^ Some prominent examples are summarized in Scheme 1. Over the years, also silicon‐based polyethers, their syntheses as well as their coordination behavior towards different Lewis acids were studied.^5^ For a long time, template‐assisted syntheses of cyclosiloxanes were only observed (accidently) by using highly reactive starting materials in the presence of silicon grease (Scheme 2).^6^, ^7^, ^8^, ^9^, ^10^ Ring transformations in the presence of metal cations were described later and the generation of sila‐polyethers by metal‐templated ring‐opening polymerization was presented as an interesting possibility to obtain novel silicon‐based ring systems.^5^, ^11^, ^12^ The coordination behavior of silicon‐based macrocycles, however, is significantly different to that of organic ethers, which is accompanied by the characteristics of the Si−O bond. Different explanations for the reduced capability of binding Lewis acids shown by siloxanes were provided in the literature.

Most recently, both concepts—covalency and ionicity—were considered as harmonious models to understand the basicity of siloxanes. Regarding a covalent model, negative hyperconjugation interactions are described in the case p(O)→σ*(Si−C).^14^, ^15^ This strengthening of the Si−O bond then competes with the shift of electron density towards Lewis acids. Concerning the ionic model, the highly polarized Si−O bond features spatially diffuse electron pairs around the O‐atom and repulsive interaction in between Si^δ+^ and metal^*n*+^ disturbs silyl ether bonding.^16^, ^17^, ^18^ Both, covalency and ionicity gain simultaneously in importance when the Si‐O‐Si angle increases and basicity lowers.^19^, ^20^, ^21^ Given that the understanding of the Si−O bond was established in this case, research in the field of coordination chemistry with cyclosiloxanes and related ligand systems showed clearly that these effects have to be taken into account. Nonetheless, the coordination chemistry turned out to be very suitable for early Group 1 and 2 metal ions, especially within disila‐ligands, which provide more suitable bite angles than cyclosiloxanes of D_*n*_ type (D=Me_2_SiO, *n*=6, 7).^22^, ^23^, ^24^, ^25^, ^26^ The nature of a cation, which shall be complexed within siloxane moieties, is preferably hard.^13^, ^27^, ^28^, ^29^, ^30^, ^31^ In the case of the early alkaline earth‐metal ions, even the commercially available cyclosiloxanes are able to dissolve salts characterized by a high lattice energy and stable complexes were obtained.^32^ Soft cations like Rb^+^ and Cs^+^ so far relucted silyl ether coordination.^33^, ^34^ Overall, sila‐ligands are suitable ligands for early s‐block‐metal coordination chemistry and opened a new chapter in host–guest chemistry over the years. In this contribution, we report the synthesis of novel hybrid disila‐crown ether moieties, which are accessible by alkaline earth metal‐template synthesis. First outlooks of a targeted template synthesis of hybrid disila‐ligands were given in past works. Scheme 2 represents sila‐ligands formed by metal templates.

Apart from siloxane coordination chemistry, the metal⋅⋅⋅O−Si interaction should also be put in context to Si−O bond activation. From the perspective of Si−O bond activation, weakening of Si−O bonds is of considerable significance regarding the conversion of silicates to silanes. Especially dicatecholato‐silicate(IV) complexes are frequently used as precursors for either silane synthesis or functionalization of various organic molecules.^35^, ^36^, ^37^, ^38^, ^39^, ^40^, ^41^, ^42^, ^43^ Most recently, a collaborative research of Ollivier and co‐workers together with Fensterbank and co‐workers demonstrated a new synthesis for ketones, in this context employing acyl chlorides.^44^

## Results and Discussion

**Scheme 3:**
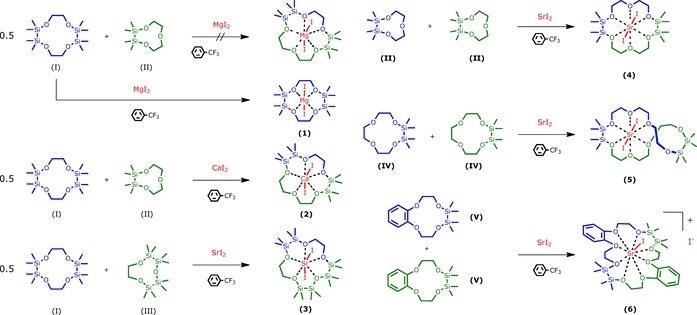
Alkaline earth metal‐template cross‐coupling reactions (left) and coupling reactions (right) of the silicon‐based crown ethers **1**–**6**.

**Figure 1:**
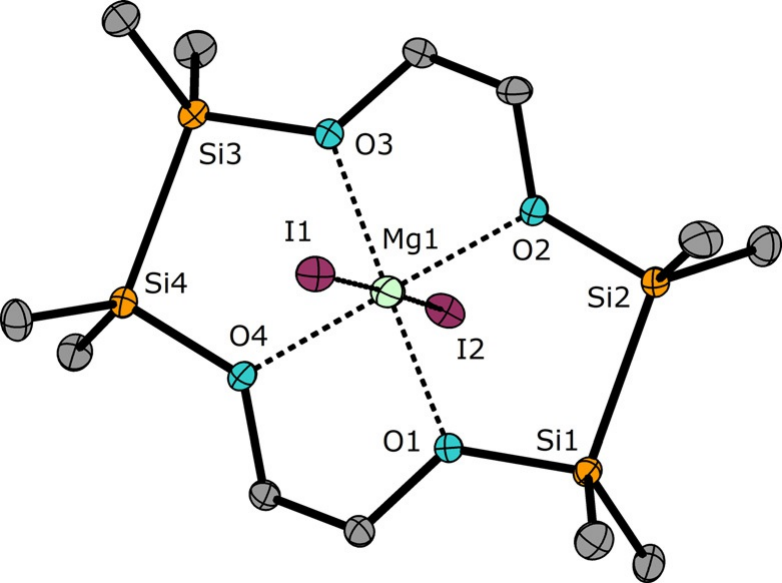
Molecular structure of **1** in the crystal. Thermal ellipsoids represent the 50 % probability level. Hydrogen atoms are omitted for clarity. Selected bond lengths [pm]: O1−Mg1 198.8(3), O2−Mg1 199.9(3), O3−Mg1 199.4(3), O4−Mg1 200.3(3), Si1−Si2 236.3(1), Si3−Si4 236.0(1). I1−Mg1 305.4(2), I2−Mg1 302.8(2). Selected bond angles [°]: O1‐Mg1‐O2 96.1(1), O3‐Mg1‐O4 98.2(1), I1‐Mg1‐I2 176.6(1).

Starting with the Mg^2+^ ion, we aimed at synthesizing a [15]crown‐5 ether by cross‐coupling the ligands 1,2,7,8‐tetrasila[12]crown‐4 (**I**) and 1,2‐disila[9]crown‐3 (**II**). However, the reaction in α,α,α‐trifluorotoluene heated at reflux failed (Scheme 3). Instead, [Mg(1,2,7,8‐tetrasila[12]crown‐4)I_2_] (**1**) was obtained and, as can be seen from the molecular structure determined by single‐crystal X‐ray diffraction analysis (SC‐XRD), the moiety of crown ether **I** is large enough to bind Mg^2+^ (Figure 1). This is probably due to the insertion of two disilane units with Si−Si bonds of 236.0(1) and 236.3(1) pm. The coordination mode of Mg^2+^ in the siloxane framework of **1** is very similar to that of [Mg(D_6_)I_2_] and the octahedral coordination towards Mg^2+^ is preferred.^32^ Also in solution, a strong interaction of ligand **I** with Mg^2+^ is present because the ^29^Si{^1^H} NMR chemical shift is observed at 22.8 ppm and thus, a strong lowfield shift in comparison with **I** (*δ*=10.8 ppm)^20^ is observed.

**Figure 2:**
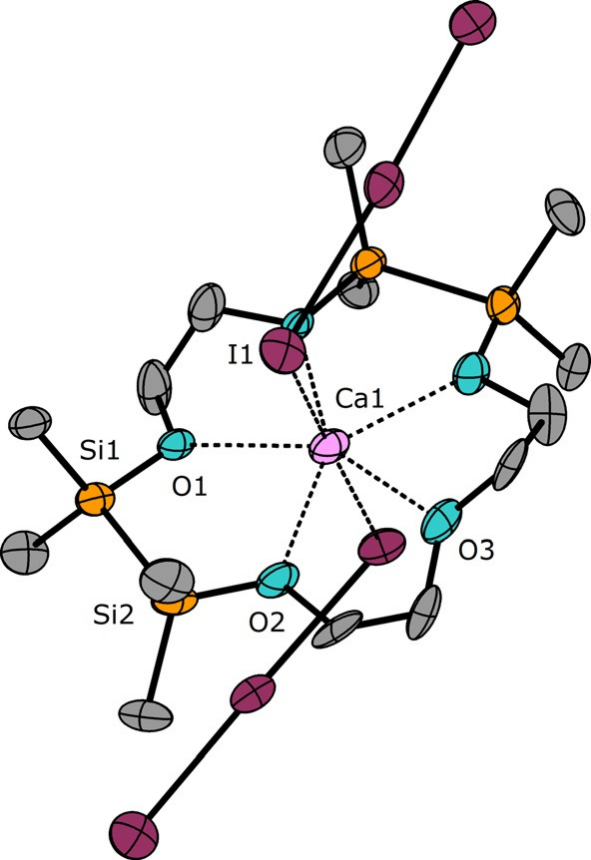
Molecular structure of **2 a** in the crystal. Thermal ellipsoids represent the 40 % probability level. Hydrogen atoms are omitted for clarity. Non‐labeled atoms are symmetry generated over 1−*x*, *y*, 1/2 −*z*. Selected bond lengths [pm]: O1−Ca1 239.3(7), O2−Ca1 245.2(9), O3−Ca1 236.4(9), Si1−Si2 234.4(3), I1−Ca1 312.6(1). Selected bond angles [°]: O1‐Ca1‐O2 77.6(2), I1‐Ca1‐I1# 177.6(1).

The fivefold, coplanar coordination by sila‐crown ligands towards the larger Ca^2+^ ion was described earlier and, as can be seen from various examples, seems to match perfectly.^26^, ^32^ Hence, we repeated the reaction of **I** and **II** with CaI_2_. After workup procedures two new signals in the ^29^Si{^1^H} NMR are observed at 19.6 and 19.9 ppm. Crystallization of the reaction product **2** however, was unsuccessful from different solvents and temperatures also due to poor solubility. We therefore expanded the anion I^−^ to I_3_^−^ upon iodine addition. The compound [Ca(1,2,7,8‐tetrasila[15]crown‐5)(I_3_)_2_] (**2 a**) then was crystallized and the molecular structure was determined by means of SC‐XRD (Figure 2). Compound **2 a** contains the new ligand 1,2,7,8‐tetrasila[15]crown‐5, which was formed by a cross‐coupling reaction of the ligands **I** and **II** mediated by the Ca^2+^ ions. The cross‐coupling of sila‐ligands is an elegant way to obtain novel macrocycles. The synthesis of these macrocycles cannot yet be realized by conventional silane chemistry and thus, the alkaline earth metal template opens up new synthetic pathways of such new ligands. The O_Si_−Ca^2+^ distances measure 239.3(7) and 245.2(9) pm and compare well to the complex [Ca(1,2‐disila[18]crown‐6)OTf_2_] (OTf=CF_3_SO_3_^−^).^26^

The synthesis of [Sr(1,2,4,5,10,11‐hexasila[15]crown‐5)I_2_] (**3**) is another example of a cross‐coupling reaction in which an alkaline earth metal cation is used. **3** represents the first hybrid disila‐crown ether bearing more disilane than ethylene units. The even larger Sr^2+^ cation can be used as a convex template to cross‐couple **I** and 1,2,4,5‐tetrasila[9]crown‐3 (**III**), which is accessible applying reaction conditions reported before.^27^ The reaction yields a silicon‐based [15]crown‐5 ether merging half an equivalent of **I** and one equivalent of **III**. Due to three disilane units within the ligand framework, the Sr^2+^ cation fits well in the cavity of the obtained ligand. With 19.44, 19.27, and 13.32 ppm, three resonances are observed in the ^29^Si{^1^H} NMR spectrum, all of which compare well to those of O_Si_⋅⋅⋅Sr^2+^ coordination compounds characterized before.^26^, ^27^ A crystal structure of **3** could not be obtained, even after iodine addition. Hence, the Lewis acidic salt GaI_3_ was added as an acceptor for the iodide anion. It is notable, that also in **3 a**, the SiSi−O−SiSi fragment of the siloxane framework provides a small Si‐O‐Si angle, which measures 121.1(6)°. The O_Si−O−Si_⋅⋅⋅Sr^2+^ distance might be the longest O⋅⋅⋅Sr^2+^ distance observed in **3 a**, but the high basicity of siloxanes towards Group 2 ions is clearly emphasized.

**Figure 3:**
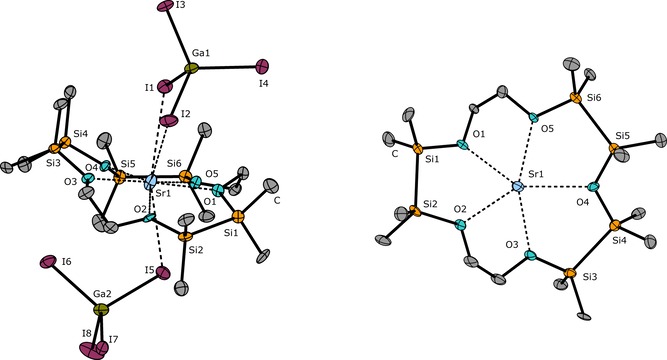
Molecular structure of **3 a** in the crystal. Contact‐ion pair (left) and top view (right). Thermal ellipsoids represent the 50 % probability level. Hydrogen atoms are omitted for clarity. Selected bond lengths [pm]: O1−Sr1 251.6(9), O2−Sr1 257.4(7), O3−Sr1 250.5(7), O4−Sr1 272.4(9), O5−Sr1 253.5(7), Si1−Si2 235.5(5), Si3−Si4 236.8(4), Si5−Si6 234.46. I1−Sr1 347.1(1), I2−Sr1 399.8(1), I5−Sr1 343.2(1). Selected bond angles [°]: O1‐Sr1‐O2 73.8(3), O3‐Sr1‐O4 79.4(3), O4‐Sr1‐O5 77.0(3).

**Figure 4:**
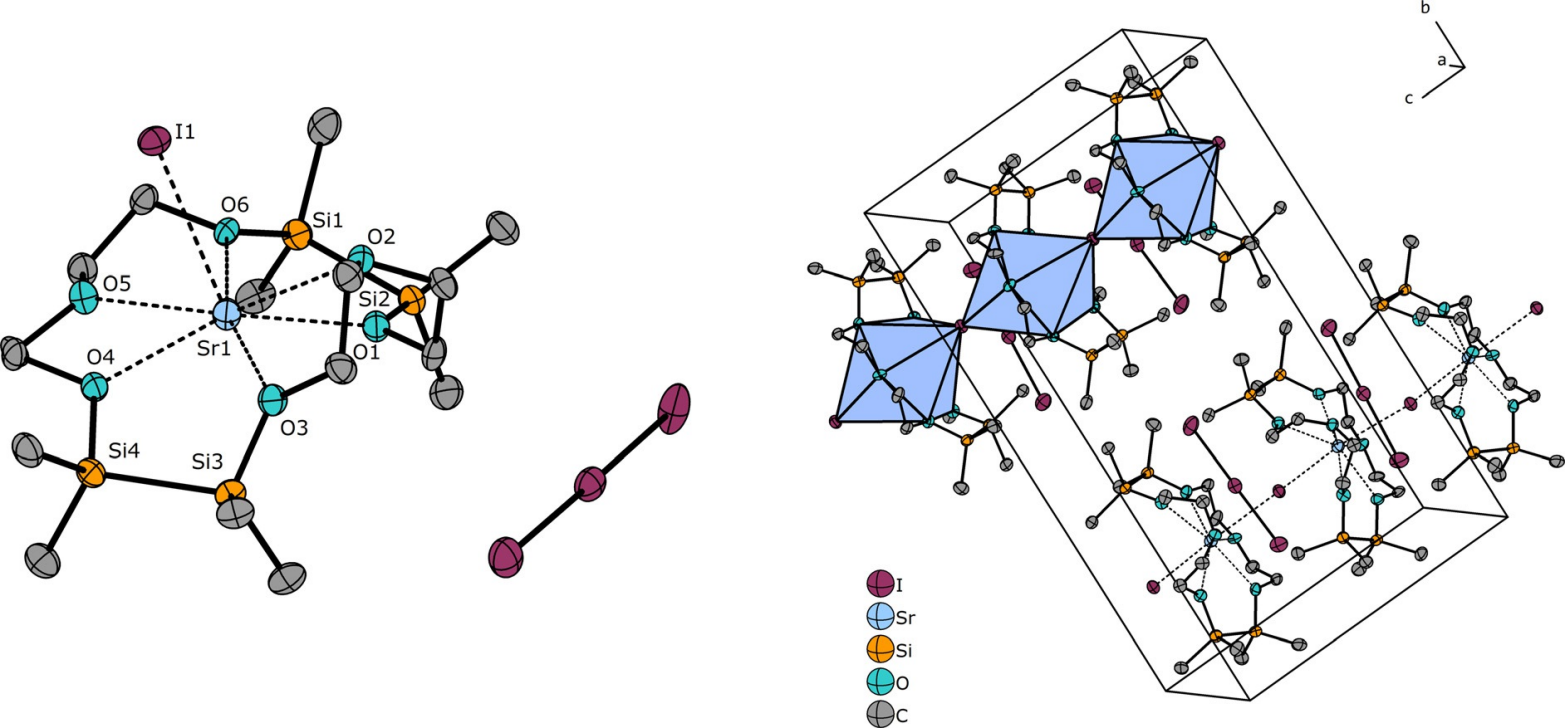
Molecular structure of **4 a** in the crystal (left) and cell package approximately along [100] (right). Thermal ellipsoids represent the 50 % probability level. Hydrogen atoms are omitted for clarity. Selected bond lengths [pm]: O1−Sr1 262.8(4), O2−Sr1 270.7(5), O3−Sr1 277.6(5), O4−Sr1 265.9(4), O5−Sr1 261.1(4), O6−Sr1 265.4(4), Si1−Si2 231.8(3), Si3−Si4 233.8(3), I1−Sr1 327.5(1). Selected bond angles [°]: O1‐Sr1‐O2 71.6(1), O3‐Sr1‐O4 69.5(1), I1‐Sr1‐O2 78.8(3).

After several months, a few single crystals of [Sr(1,2,4,5,10,11‐hexasila[15]crown‐5)(GaI_4_)_2_] (**3 a**) were obtained, which were analyzed through SC‐XRD (Figure 3). The O_Si_⋅⋅⋅Sr^2+^ distances (concerning Si−O−C as well as Si−O−Si donor groups) compare well to various complexes of Sr^2+^ and sila ligands, which were reported before.^26^, ^27^ Using the Sr^2+^ cation as a template, we were able to observe reactions of other disila‐crown ethers as well. The small cavity of **II** does not allow for 1:1 complexation of Sr^2+^ and thus, a total of two equivalents of **II** react to form the 1,2,10,11‐tetrasila[18]crown‐6 ether. [Sr(1,2,10,11‐tetrasila[18]crown‐6)I_2_] (**4**) is obtained as a colorless powder and in solution, a resonance at 17.5 ppm in the ^29^Si{^1^H} NMR spectrum was observed. Given that we were also experiencing problems with the crystallization of this compound, the X‐Ray structure could only be determined upon iodine addition. According to a reduced niggli formula, compound 1∞ [Sr(1,2,10,11‐tetrasila[18]crown‐6I_2/2_]I_3_ (**4 a**) was obtained as brown platelets which were investigated through SC‐XRD. As shown by the molecular structure in the crystal, the ligand does not perfectly match with the Sr^2+^ cation because a coplanar arrangement of the donor atoms is not observed (see Figure 4). However, all oxygen atoms of the crown ether still manage to participate in the coordination. Upon iodine addition, one I_3_^−^ anion is formed, which does not coordinate the central ion in the solid state. This enables the formation of [Sr(1,2,10,11‐tetrasila[18]crown‐6)I]^+^ fragments to build infinite chains along [001] which is most likely the driving force for crystallization. The O_Si_⋅⋅⋅Sr^2+^ distances in **4 a** compare well to those in compound **3 a** and [Sr(1,2‐disila[18]crown6)I_2_].^24^

**Scheme 4:**
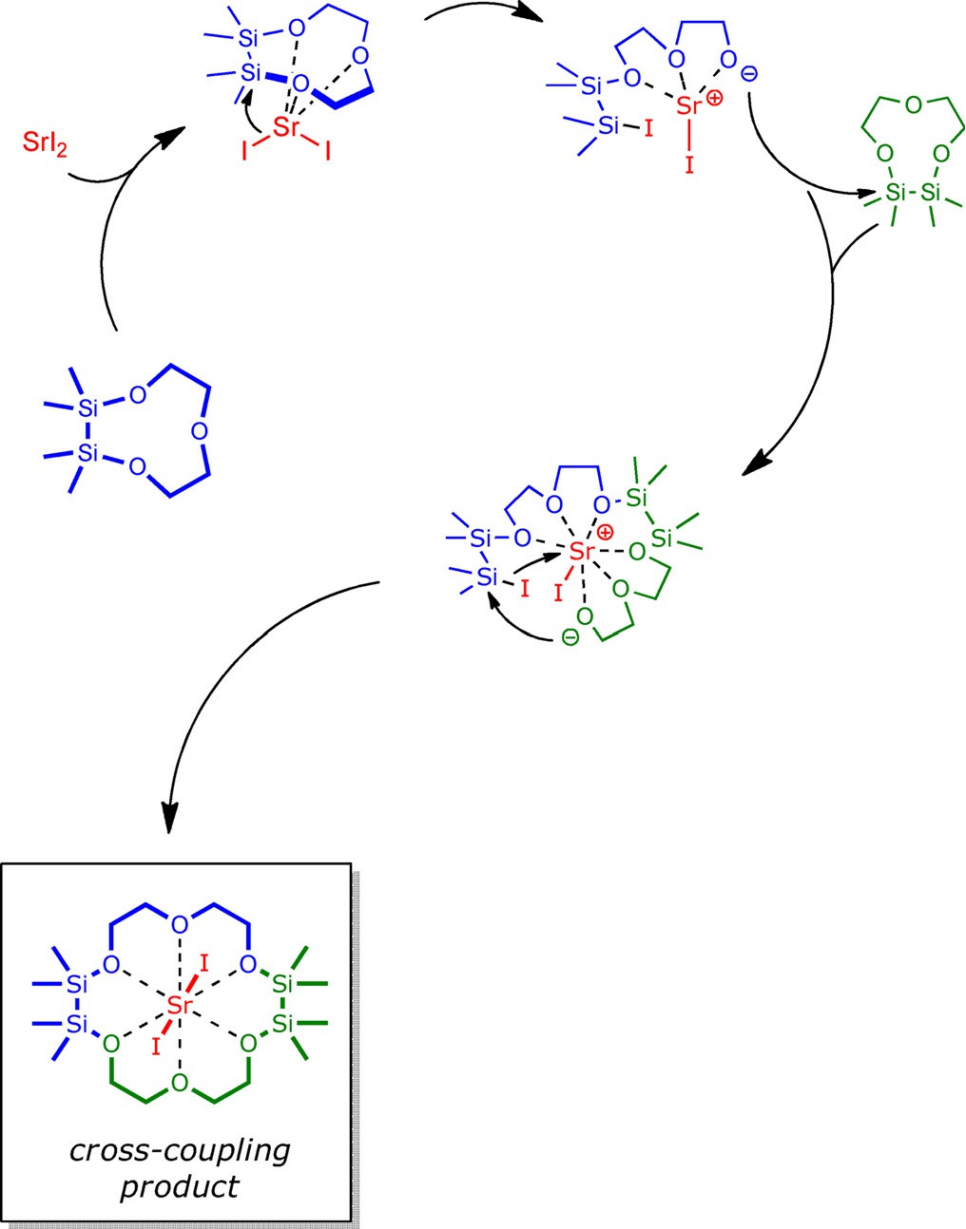
Proposed mechanism for the formation of compound **4**.

At this point, it is clear that (cross‐)coupling reactions are possible with different alkaline earth metal iodides. We tried to understand how the reaction works and the mechanism behind the formation of these unprecedented macrocycles, but unfortunately, we were unable to characterize any intermediate products by means of NMR spectroscopy or SC‐XRD. We can assure that heating at reflux is needed to cleave the smaller rings. Further, we can assure that the reaction does only occur if an iodide salt is employed. Other alkaline earth metal halides do not form these ligands. This lets us conclude that one key step in the formation of the (cross‐)coupling product has to be a nucleophilic substitution reaction. Furthermore, as evident from different works, we could convincingly show that the Si−O bond is significantly weakened due to coordinating an alkaline earth metal ion. Thus, the Si−O bond instead of the C−O bond is cleaved, which is also represented by the herein obtained macrocycles. A proposed mechanism for the formation of the cross coupling products is depicted in Scheme 4 for **4** as an example. As drawn here, the sila‐ligand is polarized by the Lewis acid first, which makes it electrophilic and thus accessible for nucleophilic attack of I^−^. Afterwards, the Si−O bond is cleaved. Subsequently, the intermediate then has to cleave the second sila‐crown. At this point of the reaction, the alkaline earth metal ion acts as a convex template, in which the open‐chained ligand species is brought into a suitable conformation for ring closure, which is the final step of the presented reactions. We want to emphasize, that such a mechanism was also postulated by Harder in a past work.^31^

**Figure 5:**
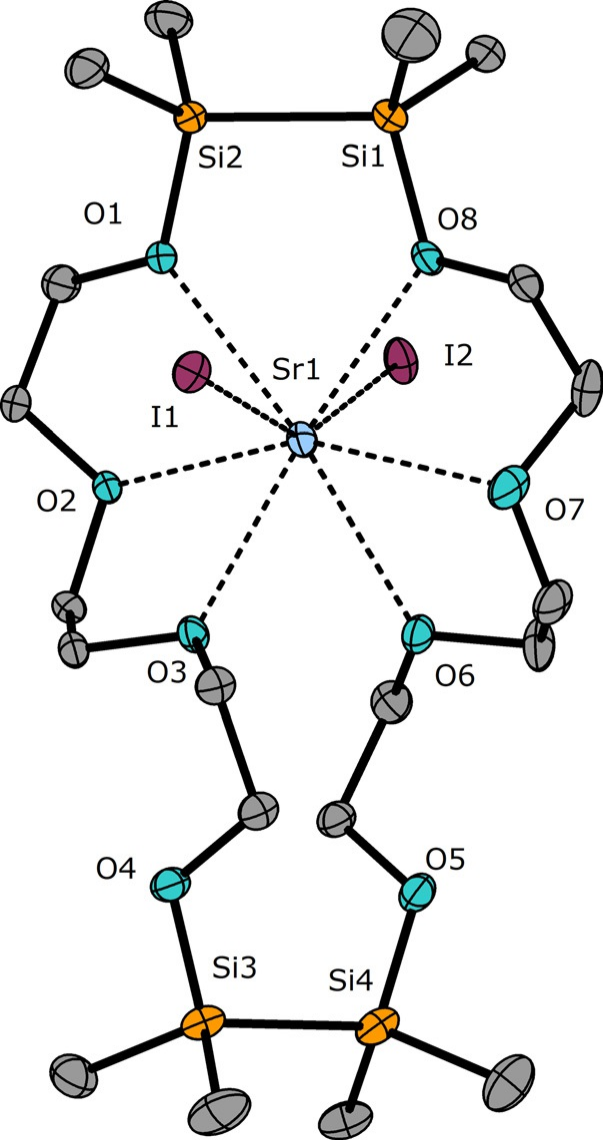
Molecular structure of **5** in the crystal. Thermal ellipsoids represent the 50 % probability level. Hydrogen atoms are omitted for clarity. Selected bond lengths [pm]: O1−Sr1 265.8(1), O2−Sr1 261.9(1), O3−Sr1 262.8(1), O4⋅⋅⋅Sr1 543.1(1), O5⋅⋅⋅Sr1 539.2(2), O6−Sr1 270.3(2), O7−Sr1 266.3(2), O8−Sr1 261.9(2), Si1−Si2 234.4(1), Si3−Si4 238.2(1), I1−Sr1 335.2(4), I2−Sr1 326.4(4). Selected bond angles [°]: O1‐Sr1‐O8 76.6(5), O3‐Sr1‐O6 67.6(1), I1‐Sr1‐I2 150.9(1).

Further reactions of SrI_2_ with the ligands 1,2‐disila[12]crown‐4 (**IV**) and 1,2‐disila‐benzo[12]crown‐4 (**V**) were performed. Both ligands are too small for Sr^2+^ and thus, template‐assisted ring opening yielded novel species by intermolecular coupling of the respective crown ether. The reaction of **IV** with SrI_2_ results in the formation of the first disilanyl‐bearing [24]crown‐8 ether. [Sr(1,2,13,14‐tetrasila[24]crown‐8)I_2_] (**5**) was obtained by coupling two equivalents of **IV** (Figure 5). The molecular structure in the crystal reveals that six out of eight oxygen atoms, as well as the two iodide anions, are coordinating. The overall eightfold coordinated Sr^2+^ cation is therefore embedded in a *pseudo*‐1,2‐disila[18]crown‐6 moiety. Given that the two oxygen atoms of the second disilane fragment do not coordinate, a twisting of the crown ether is observed. The driving force of this reaction is the arrangement of six of the coordinating crown ether oxygen atoms forming an [18]crown‐6‐like coordination sphere similar to that observed in **4**. The O_Si_⋅⋅⋅Sr^2+^ distances in **5** are, however, slightly shorter than those in [Sr(1,2‐disila[18]crown6)I_2_].^26^ A single ^29^Si{^1^H} NMR resonance signal for **5** is observed at 15.2 ppm. A split of this resonance at low temperature of 190 K was not observed.

Thus, the exchange between the coordinating disilane units is too fast on the NMR time scale and results in the described equivalency. At this point, it should be noted that the spectroscopic investigation of the compound is challenging. Compound **5** decomposes readily with only traces of moisture forming [Sr(11,12‐disila‐EO7)I]I (EO7=heptaethylene glycol).^13^

**Figure 6:**
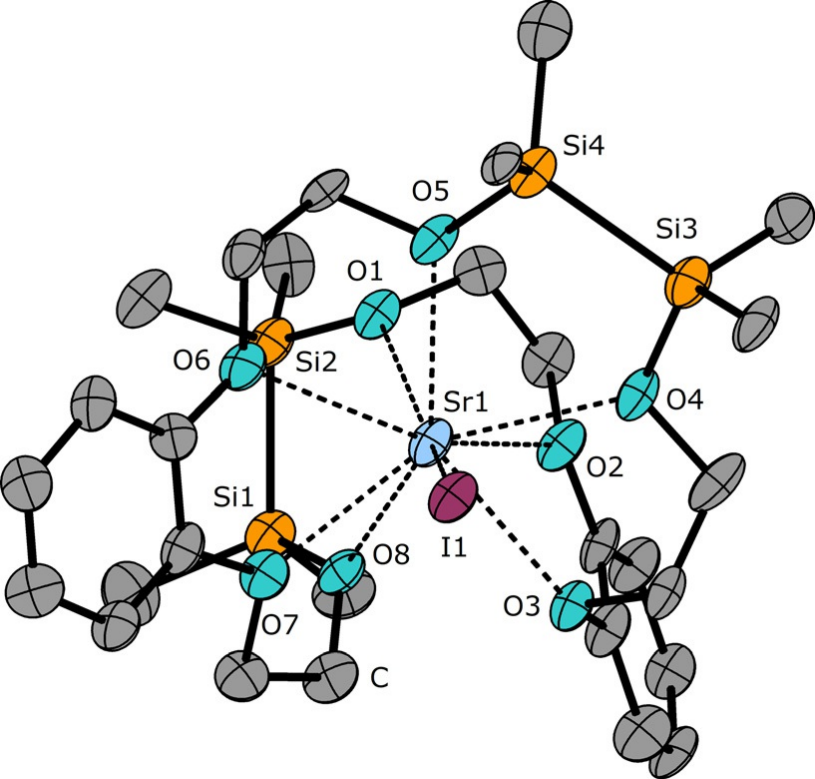
Molecular structure of the cationic part of **6** in the crystal. Thermal ellipsoids represent the 40 % probability level. Hydrogen atoms as well as a single iodide anion are omitted for clarity. Selected bond lengths [pm]: O1−Sr1 265.0(10), O2−Sr1 274.1(8), O3−Sr1 266.0(9), O4−Sr1 263.9(9), O5−Sr1 260.8(10), O6−Sr1 270.1(8), O7−Sr1 263.8(10), O8−Sr1 271.9(9), Si1−Si2 234.6(7), Si3−Si4 234.8(5), I1−Sr1 329.9(1). Selected bond angles [°]: O4‐Sr1‐O5 72.8(3), O6‐Sr1‐O7 58.4(3).

The twisting of the crown ether is most likely a suitable preorganization for the formation of the open‐chained EO7 ligand and explains its high sensitivity. [Sr(1,2,13,14‐tetrasila‐dibenzo[24]crown‐8)I]I (**6**) is more stable and was characterized by state‐of‐the‐art methods including SC‐XRD (Figure 6). After a 2:1 reaction of **V** with SrI_2_, **V** performs intermolecular coupling as well. Here, the dibenzo crown ether helically encapsulates the central ion under replacement of one iodide anion. All eight oxygen atoms do now participate in the coordination of Sr^2+^. Even though the ligand in **6** is more rigid than that of **5**, shielding of the central ion is observed. This might be a result of slightly reduced basicity of benzo crown ethers in general. The replacement of iodide by oxygen donor groups is favored over metal–anion interactions and is most likely also present in solution because the ^29^Si{^1^H} NMR chemical shift is with 19.8 ppm very distinct for Sr^2+^ coordination.

One of the remaining questions is of course whether it is possible to remove the metal center for possible use of such new ligand in coordination chemistry. We are currently investigating this subject, but are still at an early stage. To give an outlook, we performed exchange reactions. As an example, an excess of **6** was reacted with [222]cryptand and indeed we were able to obtain free ligand 1,2,13,14‐tetrasila‐dibenzo[24]crown‐8 (**7**) after workup. The metal‐free ligand species was characterized by means of HR‐ESI MS as well as NMR spectroscopy. In HR‐ESI^+^ MS, *m*/*z* relations of 625.2502 [**7**+H]^+^ (100) as well as 647.2320 [**7**+Na]^+^ (100) were found. In addition, ^29^Si{^1^H} NMR spectroscopy revealed highfield chemical shift and a single resonance at 11.5 ppm is found. This value compares well to the free ligand **V** and also to related metal‐free disila‐ligand systems.^22^, ^24^, ^26^, ^28^, ^33^ The problem so far is that a significant amount of the cryptand remains in the oily residue after conversion, so no further coordination chemistry could be performed yet. To which extent this will be possible, not only for the ligands presented herein, is of current interest and will be subject of further research.

So far, we also tried (cross)‐coupling reactions with BaI_2,_ or other alkaline earth metal halides such as chlorides and bromides but various attempts failed. The method obtaining the novel macrocycles herein is for this reason restricted to iodide salts.

## Conclusions

In this study alkaline earth metal iodides were successfully used for the coupling reaction of different silicon‐based crown ether analogues to form novel ligand environments. The work presents Si−O bond‐cleavage reactions driven by a macrocyclic effect due to metal‐template reaction of the respective silicon‐based ligands. Given that the Mg^2+^ ion is too small to cross‐couple small silicon‐based ligands, a cross‐coupling reaction was established with the larger Ca^2+^ and Sr^2+^ ions. Furthermore, the Sr^2+^ ion was used for coupling reactions of three different disila‐crown ethers. Barium as a template for coupling reaction, however, turned out to be unsuccessful. Although the organic [12]crown‐4 is too small for the Mg^2+^ ion, the cavity of the disilane‐bearing analogue 1,2,7,8‐tetrasila[12]crown‐4 (**I**) ether matches fine with the Mg^2+^ ion and the complex [Mg(1,2,7,8‐tetrasila12crown‐4)I_2_] (**1**) was obtained. [Ca(1,2,7,8‐tetrasila[15]crown‐5)I_2_] (**2**) and [Sr(1,2,4,5,10,11‐hexasila[15]crown‐5)I_2_] (**3**) were synthesized by cross‐coupling reaction of the small rings **I** and **II** for **2**, and **I** and **III** for **3**. The ligand in **3** represents the first crown ether with more disilane than ethylene units between the donor atoms. Finally, [Sr(1,2,10,11‐tetrasila[18]crown‐6)I_2_] (**4**), [Sr(1,2,13,14‐tetrasila[24]crown‐8)I_2_] (**5**) and [Sr(1,2,13,14‐tetrasila‐dibenzo[24]crown‐8)I]I (**6**) were characterized and obtained by template‐driven dimerization of **II**, **IV**, or **V** with SrI_2_. The ligand moieties observed in **2**–**6** cannot yet be synthesized by conventional silane chemistry and for this reason, the template‐assisted (cross‐)coupling of various ligands is an elegant way to obtain novel macrocycles and/or their metal complexes. Overall, this work gives an outlook on Group 2 ion‐catalyzed silane syntheses, especially due to the fact that first attempts showed that the metal ion can, in principle, be removed from a novel sila‐ether. We also hope that there will be a broader application of the presented Si−O bond activations as a mean of molecule functionalization.

## Experimental Section

### General

All manipulations were carried out with rigorous exclusion of oxygen and moisture using basic Schlenk techniques establishing an inert‐gas atmosphere with a vacuum line. All solvents were dried and freshly distilled before use. The alkaline earth metal salts MgI_2_ (Alfa Aesar, 99,996 %), CaI_2_ and SrI_2_ (Alfa Aesar, 99 %), BaI_2_ (abcr, 99.995 %) and GaI_3_ (abcr, 99 %) were finely ground and stored in a Mbraun glovebox under Ar. NMR spectra were recorded on a Bruker AV III HD 300 MHz or AV III 500 MHz spectrometer, respectively. Infrared (IR) spectra of the respective samples were measured using attenuated total reflectance (ATR) mode on the Bruker‐type spectrometer Alpha FT‐IR. ESI mass spectra were acquired with a LTQ‐FT Ultra mass spectrometer (Thermo Fischer Scientific) and LIFDI mass spectra were acquired on a JEOL AccuTOF‐GCv device. The resolution was set to 100.000. Elemental analysis was carried out on a Vario MicroCube. In case of complex **2** and **3**, we were not able to obtain an accurate elemental analysis. This is probably due to the wax‐like, greasy characteristics of the compounds and/or formation of SiC during the measurement. The crown‐ethers **I**, **II**, **IV**, and **V** were synthesized according to literature‐known procedures.^22^, ^28^, ^33^ Compound **7** can only be obtained with major impurities of [222]crypt.

### Synthesis of 1,2,4,5‐tetrasila[9]crown‐3 (III)

Ethylene glycol (0.19 mL, 3.41 mmol, 1.00 equiv) and NEt_3_ (0.94 mL, 6.84 mmol, 2.0 equiv) were dissolved in THF (50 mL). Subsequently, O(Si_2_Me_4_Cl)_2_ 23 (1 mL, 3.41 mmol, 1.00 equiv) dissolved in THF (50 mL) was added over a period of 60 min. The resulting white suspension was then stirred overnight and the solvent was removed in vacuo. The residue was extracted with *n*‐pentane (50 mL) followed by filtration. Removing the solvent under reduced pressure yielded the crown ether as a colorless oil (0.74 g, 70 %).

^1^H NMR (300 MHz, CD_3_CN): *δ*=0.18 (s, 12 H, Si(C*H*_3_)_2_), 0.20 (s, 12 H, Si(C*H*_3_)_2_), 3.69 ppm (s, 4 H, C*H*_2_); ^13^C{^1^H} NMR (125 MHz, CD_3_CN): *δ*=0.1 (s, Si(*C*H_3_)_2_), 2.8 (s, Si(*C*H_3_)_2_), 66.0 ppm (s, CH_2_); ^29^Si{^1^H} NMR (60 MHz, CD_3_CN): *δ*=1.5 (s, *Si*(CH_3_)_2_), 11.4 ppm (s, *Si*(CH_3_)_2_). HR‐MS: ESI(+) *m*/*z* (%): 309.1196 [M+H]^+^ (100), 639.2143 [2 m+Na]^+^ (100); IR: 2951 (m), 2896 (w), 2867 (w), 1457 (vw), 1388 (vw), 1247 (s), 1140 (m), 1089 (s), 1027 (s), 927 (m), 854 (m), 795 (s), 761 (vs.), 721 (m), 681 (m), 660 (m), 633 (m), 553 (m), 506 (vw), 487 cm^−1^ (m).

### Synthesis of [Mg(1,2,7,8‐tetrasila[12]crown‐4)I_2_] (1)

Compound **I** (0.100 g, 0.29 mmol) was dissolved in α,α,α‐trifluorotoluene (10 mL). Subsequently, MgI_2_ (0.079 g, 0.29 mmol) was added. Stirring the mixture at 60 °C for 90 min gave a white suspension. Removing the solvent under reduced pressure yielded a white precipitate which was extracted with DCM (10 mL) followed by filtration. After washing with *n*‐pentane (4 mL), and drying in vacuo, **1** was obtained as a colorless powder (0.139 g, 77 %). For single‐crystal growth, the powder was dissolved in DCM (4 mL) and layered with *n*‐pentane (20 mL). Single crystals were obtained overnight as colorless needles.

^1^H NMR (300 MHz, CD_2_Cl_2_): *δ*=0.59 (s, 24 H, Si(C*H*_3_)_2_), 4.23 ppm (s, 8 H, C*H*_2_); ^13^C{^1^H} NMR (125 MHz, CD_2_Cl_2_): *δ*=−0.46 (s, Si(*C*H_3_)_2_), 62.8 ppm (s, CH_2_); ^29^Si{^1^H} NMR (60 MHz, CD_2_Cl_2_): *δ*=22.8 ppm (s, *Si*(CH_3_)_2_). MS: ESI(+) *m*/*z* (%): 503.0270 [M−I]^+^ (15); IR: 2949 (vw), 2888 (vw), 1450 (w), 1398 (vw), 1369 (vw), 1247 (m), 1103 (m), 1056 (s), 1022 (s), 934 (vs.), 915 (s), 868 (m), 840 (m), 818 (s), 802 (s), 780 (vs.), 715 (s), 645 (m), 464 cm^−1^ (m). CHN calcd for C_12_H_32_I_2_MgO_4_Si_4_⋅ CH_2_Cl_2_ C, 21.81; H, 4.79. Found C, 21.66; H, 4.90.

### Synthesis of [Ca(1,2,7,8‐tetrasila[15]crown‐5)I_2_] (2)

Compound **I** (0.156 g, 0.44 mmol) together with **II** (0.195 g, 0.88 mmol) were dissolved in α,α,α‐trifluorotoluene (10 mL). Subsequently, CaI_2_ (0.259 g, 0.88 mmol) was added. Heating the mixture at reflux for 90 min resulted in a white suspension. Removing the solvent under reduced pressure yielded a white precipitate which was extracted with of DCM (10 mL) followed by filtration. After washing with two portions of *n*‐pentane (4 mL each), and drying in vacuo, **1** was obtained as a colorless powder (0.316 g, 52 %). For single‐crystal growth, **2** (30 mg, 0.04 mmol) and I_2_ (22 mg, 0.09 mmol) were dissolved in DCM (4 mL) and filtered. The filtrate was then layered with *n*‐pentane (20 mL). A few brown platelets of **2 a** were obtained after more than two weeks at ambient temperature.

^1^H NMR (300 MHz, CD_2_Cl_2_): *δ*=0.47 (s, 12 H, SiC*H*_3_), 0.48 (s, 12 H, SiC*H*_3_), 3.95–4.03 ppm (m, 12 H, C*H*_2_); ^13^C{^1^H} NMR (75 MHz, CD_2_Cl_2_): *δ*=−0.7 (s, Si*C*H_3_), −0.7 (s, Si*C*H_3_), 63.2 (s, *C*H_2_), 64.8 (s, *C*H_2_), 71.2 ppm (s, *C*H); ^29^Si{^1^H} NMR (60 MHz, CD_2_Cl_2_): *δ*=19.6 (s, *Si*CH_3_), 19.9 ppm (s, *Si*CH_3_). MS: ESI(+) *m*/*z* (%): 419.1521 [M−CaI_2_+Na]^+^ (100), 397.1705 [M−CaI_2_+H]^+^ (5). IR: 2947 (m), 2881 (w), 1455 (vw), 1400 (vw), 1248 (s), 1057 (s), 1029 (s), 943 (s), 795 (s), 768 (vs.), 723 (s), 635 (m), 505 cm^−1^ (vw).

### Synthesis of [Sr(1,2,7,8,10,11‐hexasila[15]crown‐5)I_2_] (3)

Compounds **I** (0.100 g, 0.29 mmol) and **III** (0.170 g, 0.55 mmol) were dissolved in α,α,α‐trifluorotoluene (10 mL). Subsequently, SrI_2_ (0.188 g, 0.56 mmol) was added. Heating the mixture at reflux for 90 min resulted in a white suspension. Removing the solvent under reduced pressure yielded a white precipitate which was extracted with DCM (10 mL), followed by filtration. After washing with two portions of *n*‐pentane (4 mL each) and drying in vacuo, **3** was obtained as a colorless, greasy solid (0.185 g, 40 %). For single‐crystal growth, product **3** (0.030 g, 0.04 mmol) and GaI_3_ (0.018 g, 0.04 mmol) were dissolved in α,α,α‐trifluorotoluene (5 mL). The suspension was stirred 30 min and gently warmed to 60 °C for 5 min. The mixture was then freed of the solvent, extracted with DCM (3 mL) and filtered. The filtrate was then concentrated until the saturation point was reached and stored at −32 °C. A few colorless platelets of **3 a** were obtained after more than four weeks.

^1^H NMR (300 MHz, CD_2_Cl_2_): *δ*=0.44 (s, 12 H, Si(C*H*_3_)_2_), 0.47 (s, 12 H, Si(C*H*_3_)_2_), 0.56 (s, 12 H, Si(C*H*_3_)_2_), 3.96 ppm (s, 8 H, C*H*_2_); ^13^C{^1^H} NMR *δ*=−0.5 (s, Si(*C*H_3_)_2_), −0.5 (s, Si(*C*H_3_)_2_), 4.1 (s, Si(*C*H_3_)_2_), 65.3 (s, CH_2_), 65.4 ppm (s, *C*H_2_); ^29^Si{^1^H} NMR (60 MHz, CD_2_Cl_2_): *δ*=13.3 (s, *Si*(CH_3_)_2_), 19.3 (s, *Si*(CH_3_)_2_), 19.4 ppm (s, *Si*(CH_3_)_2_). MS: ESI(+) *m*/*z* (%): 698.9902 [M−I]^+^ (100), 507.1697 [M−SrI_2_+Na]^+^ (30). IR: 2960 (w), 2879 (vw), 1456 (vw), 1400 (vw), 1369 (vw), 1258 (s), 1074 (s), 1024 (s), 951 (s), 894 (m), 851 (m), 794 (vs.), 769 (vs.), 712 (s), 634 (m), 557 (m), 497 (w), 448 cm^−1^ (w).

### Synthesis of [Sr(1,2,10,11‐tetrasila[18]crown‐6)I_2_] (4)

Compound **II** (0.258 g, 1.17 mmol) was dissolved in α,α,α‐trifluorotoluene (10 mL). Subsequently SrI_2_ (0.200 g, 0.59 mmol) was added. Heating the mixture at reflux for 60 min. resulted in a white suspension. Removing the solvent under reduced pressure gave a white precipitate which was extracted with DCM (20 mL) followed by filtration. Upon washing with *n*‐pentane (5 mL) and removal of the solvent, **4** was obtained as a pale white powder (0.259 g, 56 %). For single crystal growth, product **4** (30 mg 0.04 mmol) and I_2_ (10 mg, 0.04 mmol) were dissolved in DCM (4 mL). Layering the solution with *n*‐pentane (20 mL) yielded single crystals of **4 a** as brown blocks after three days.

^1^H NMR (300 MHz, CD_2_Cl_2_): *δ*=0.45 (s, 12 H, SiC*H*_3_), 3.88 (t, ^3^ *J*_HH_=4.6 Hz, 8 H, C*H*_2_), 4.08 ppm (t, ^3^ *J*_HH_=4.6 Hz, 8 H, C*H*_2_); ^13^C{^1^H} NMR (125 MHz, CD_2_Cl_2_): *δ*=0.1 (s, Si*C*H_3_), 63.0 (s, *C*H_2_), 72.8 ppm (s, *C*H_2_); ^29^Si{^1^H} NMR (60 MHz, CD_2_Cl_2_): *δ*=17.5 ppm (s, *Si*CH_3_). MS: ESI(+) *m*/*z* (%): 264.0475 [M−2I]^2+^ (93), 447.2058 [M−SrI_2_+Li]^+^ (3), 463.1797 [M−SrI_2_+Na]^+^ (55), 479.1537 [M−SrI_2_+K]^+^ (15), 655.0003 [M−I]^+^ (16). IR: 2943 (w), 2880 (w), 1459 (w), 1398 (vw), 1353 (w), 1247 (m), 1069 (s), 1039 (s), 944 (s), 927 (s), 861 (s), 840 (s), 819 (s), 795 (s), 768 (vs.), 718 (s), 629 (m), 546 (w), 523 cm^−1^ (w). CHN calcd for C_16_H_40_I_2_O_6_Si_4_Sr: C, 24.57; H, 5.15. Found: C, 25.42; H, 5.35.

### Synthesis of [Sr(1,2,13,14‐tetrasila[24]crown‐8)I_2_] (5)

Compound **IV** (0.209 g, 0.79 mmol) was dissolved in α,α,α‐trifluorotoluene (15 mL). Subsequently SrI_2_ (0.135 g, 0.40 mmol) was added. Heating the mixture at reflux for three hours resulted in a white suspension. Removing the solvent under reduced pressure yielded a white precipitate which was extracted with DCM (20 mL), followed by filtration. The product **5** was then obtained as a colorless powder after removal of the solvent (0.252 g, 74 %). For single‐crystal growth, the powder was dissolved in DCM (4 mL) and layered with *n*‐pentane (20 mL). Single crystals were obtained overnight as colorless needles.

^1^H NMR (300 MHz, CD_2_Cl_2_): *δ*=0.37 (s, 24 H, Si(C*H*_3_)_2_), 3.94 (s, 8 H, C*H*_2_), 3.95 ppm (s, 16 H, C*H*_2_); ^13^C{^1^H} NMR (125 MHz, CD_2_Cl_2_): *δ*=−0.09 (s, Si*C*H_3_), 62.8 (s, *C*H_2_), 70.5 (s, *C*H_2_), 73.5 ppm (s, *C*H_2_); ^29^Si{^1^H} NMR (60 MHz, CD_2_Cl_2_): *δ*=15.2 ppm (s, *Si*CH_3_). MS: ESI(+) *m*/*z* (%): 551.2311 [M−SrI_2_+Na]^+^ (82); IR: 2961 (m), 2943 (m), 2882 (w), 1454 (w), 1396 (w), 1366 (w), 1349 (w), 1302 (w), 1259 (s), 1091 (s), 1073 (s), 1039 (s), 1017 (s), 951 (s), 931 (s), 861 (m), 840 (m), 791 (vs.), 764 (s), 732 (s), 661 (s), 636 (m), 563 (w), 492 (w), 424 cm^−1^ (w). CHN calcd for C_20_H_48_I_2_O_8_Si_4_Sr: C, 27.86; H, 5.67. Found: C, 27.60; H, 5.67.

### Synthesis of [Sr(1,2,13,14‐tetrasila‐dibenzo[24]crown‐8)I]I (6)

Compound **V** (0.200 g, 0.64 mmol) was dissolved in α,α,α‐trifluorotoluene (15 mL). Subsequently, SrI_2_ (0.109 g, 0.32 mmol) was added. Heating the mixture at reflux for three hours resulted in a white suspension. Removing the solvent under reduced pressure yielded a white precipitate which was extracted with DCM (20 mL) followed by filtration. The product **6** was then obtained as a colorless powder after removal of the solvent (0.200 g, 65 %). For single‐crystal growth, the powder was dissolved in DCM (3 mL) and layered with *n*‐pentane (20 mL). Single crystals were obtained overnight as colorless platelets.

^1^H NMR (300 MHz, CD_2_Cl_2_): *δ*=0.45 (s, 24 H, SiC*H*_3_), 4.15 (t, 8 H, C*H*_2_, ^3^ *J*_HH_=4.6 Hz), 4.42 (t, 8 H, C*H*_2_, ^3^ *J*_HH_=4.6 Hz), 7.02–7.16 ppm (m, 8 H, C*H*_Ar_); ^13^C{^1^H} NMR (125 MHz, CD_2_Cl_2_): *δ*=0.0 (s, Si*C*H_3_), 62.4 (s, *C*H_2_), 72.6 (s, *C*H_2_), 116.1 (s, *C*H_Ar_), 124.9 (s, *C*H_Ar_), 147.4 ppm (s, *C_q_*H_Ar_); ^29^Si{^1^H} NMR (60 MHz, CD_2_Cl_2_): *δ*=19.8 ppm (s, *Si*CH_3_). MS: ESI(+) *m*/*z* (%): 647.2311 [M−SrI_2_+Na]^+^ (78), 839.0525 [M−I]^+^ (35); IR: 2946 (w), 2885 (w), 1595 (w), 1500 (s), 1456 (m), 1400 (w), 1366 (w), 1250 (vs.), 1191 (m), 1161 (m), 1115 (m), 1063 (s), 934 (vs.), 916 (vs.), 862 (m), 837 (m), 817 (s), 774 (s), 750 (vs.), 734 (s), 711 (s), 630 (w), 605 (w), 533 (w), 516 (w), 484 (w), 456 (w), 422 cm^−1^ (w). CHN calcd for C_28_H_48_I_2_O_8_Si_4_Sr⋅0.5 CH_2_Cl_2_: C, 33.93; H, 4.90. Found: C, 33.92; H, 4.68.

### Synthesis of 1,2,13,14‐tetrasila‐dibenzo[24]crown‐8 (7)

Compound **6** (0.190 g, 0.19 mmol, excess) was dissolved in DCM (10 mL) and [222]cryptand (0.050 mg, 0.13 mmol, 0.7 equiv) was added. The resulting suspension was stirred overnight to give a clear solution. The solvent was removed under reduced pressure to give an oily, greasy residue. Extracting with *n*‐pentane (10 mL) and subsequent filtering of the suspension yielded a clear solution. Removing the solvent under reduced pressure gave a greasy crown ether‐[222]cryptand mixture (0.13 g). [222]crypt contamination was more than 30 %.

^1^H NMR (300 MHz, CD_2_Cl_2_): *δ*= 0.24 (s, 24 H, SiC*H*_3_), 3.96–4.01 (m, 8 H, C*H*_2_), 4.02–4.08 (m, 8 H, C*H*_2_), 6.91 ppm (m, 8 H, C*H*_AR_); ^13^C{^1^H} NMR (125 MHz, CD_2_Cl_2_): *δ*= 0.8 (s, Si*C*H_3_), 63.5 (s, *C*H_2_), 72.5 (s, *C*H_2_), 116.7 (s, *C*_AR_), 122.5 (s, *C*_AR_), 150.5 ppm (s, *C*_ARq_); ^29^Si{^1^H} NMR (60 MHz, CD_2_Cl_2_): *δ*= 11.5 ppm (s, *Si*CH_3_). MS: HR‐ESI(+) *m*/*z* (%): 625.2502 [M+H]^+^ (100), 647.2320 [M+Na]^+^ (100). IR: 2945 (w), 2869 (w), 1593 (w), 1501 (s), 1452 (m), 1396 (w), 1370 (w), 1328 (w), 1247 (s), 1221 (m), 1124 (s), 1098 (s), 1053 (s), 948 (m), 923 (m), 823 (m), 790 (s), 763 (vs.), 740 (s), 631 (m), 450 cm^−1^ (w).

### Crystallography

Single‐crystal X‐ray diffraction experiments were carried out on a Bruker D8 Quest (**3 a**, **5**), STOE IPDS2 (**1**⋅DCM, **6**), STOE IPDS2T (**2 a**) or a STOE STADIVARI (**4 a**) diffractometer, respectively. Measurements were performed at 100 K with MoKα (*λ*=0.71073 Å) or CuKα (*λ*=1.54184 Å) radiation, graphite monochromatization or respective X‐ray optics. The structures were solved by direct methods and refinement with full‐matrix‐least‐squares against *F* ^2^ using *SHELXT* and *SHELXL* on *OLEX2* platform.^45^, ^46^, ^47^ The crystallographic data for all compounds is deposited in the Cambridge Crystallographic Data Centre (CCDC).^51^ Due to severe disorder of solvent molecules in the crystal structure of **3 a** and **6**, we applied the SQUEEZE operation. A selection of respective crystal data is given below.

**Crystal data of 1⋅DCM**: C_13_H_34_Cl_2_I_2_MgO_4_Si_4_, monoclinic, *P*2_1_, *Z*=2, 100(2) K, *a*=8.173(3), *b*=12.204(4), *c*=14.204(8) Å; *β*=93.02(4)°, *V=*1414.8(11) Å^3^, *ρ*=1.680 g cm^−3^, numerical absorption correction using STOE X‐AREA and X‐RED32.^48^ *μ*=2.618 mm^−1^, *T*_min_, *T*_max_=0.5303, 0.8053, 2*Θ* range 4.402–58.996°, reflections measured 21 645, independent reflections 7891 [R(int)=0.0451, 244 parameters, R‐index [*I*≥2*σ*(*I*)] 0.0244, w*R*_2_ (all data) 0.0590, GoF 1.047, Δ*ρ*_max_, Δ*ρ*_min_ 1.27/−0.52 e Å^3^.

**Crystal data of 2 a**: C_14_H_36_CaI_6_O_5_Si_4_, monoclinic, *C*2/*c*, *Z*=4, 100(2) K, *a*=11.408(3), *b*=12.970(2), *c*=25.570 Å; *β*=92.38(2)°, *V=*3484.7(16) Å^3^, *ρ*=2.284 g cm^−3^, spherical absorption correction using STOE X‐AREA and LANA.^49^ *μ*=5.654 mm^−1^, *T*_min_, *T*_max_=0.1989, 0.4381, 2*Θ* range 3.458–50.994°, reflections measured 13 999, independent reflections 3250 [R(int)=0.0924], 154 parameters, R‐index [*I*≥2*σ*(*I*)] 0.0548, w*R*_2_ (all data) 0.1400, GoF 1.072, Δ*ρ*_max_, Δ*ρ*_min_ 1.56/−0.91 e Å^3^.

**Crystal data of 3 a**: C_16_H_44_Ga_2_I_8_O_5_Si_6_Sr, monoclinic, *P*2_1_/*c*, *Z*=4, 100(2) K, *a*=23.3580(16), *b*=11.3585(7), *c*=20.6920(14) Å; *β*=111.998(4)°, *V=*5090.2(6) Å^3^, *ρ*=2.254 g cm^−3^, spherical absorption correction using STOE X‐AREA and LANA,^49^ *μ*=7.110 mm^−1^, *T*_min_, *T*_max_=0.4793, 0.7452, 2*Θ* range 4.232–50.738°, reflections measured 171 072, independent reflections 9287 [R(int)=0.1514], 355 parameters, R‐index [*I*≥2*σ*(*I*)] 0.0675, *wR*_2_ (all data) 0.1863, GoF 1.076, Δ*ρ*_max_, Δ*ρ*_min_ 4.17/−1.57 e Å^3^.

**Crystal data of 4 a**: C_16_H_40_I_4_O_6_Si_4_Sr, monoclinic, *P*2_1_/*c*, *Z*=4, 100(2) K, *a*=12.0422(5), *b*=22.2125(6), *c*=12.8413(4) Å; *β*=95.734(3)°, *V=*3417.7(2) Å^3^, *ρ*=2.014 g cm^−3^, spherical absorption correction using STOE X‐AREA and LANA,^49^ *μ*=32.123 mm^−1^, *T*_min_, *T*_max_=0.212, 0.360, 2*Θ* range 7.378–50.994°, reflections measured 26 969, independent reflections 6337 [R(int)=0.0854], 288 parameters, R‐index [*I*≥2*σ*(*I*)] 0.0391, w*R*_2_ (all data) 0.0894, GoF 0.804, Δ*ρ*_max_, Δ*ρ*_min_ 1.23/−1.51 e Å^3^.

**Crystal data of 5**: C_20_H_48_I_2_O_8_Si_4_Sr, triclinic, *P*‐1, *Z*=2, 110(2) K, *a*=8.4870(4), *b*=14.9819(7), *c*=15.4929(7) Å, *α*=105.457(2)°, *β*=102.187(2)°, *γ*=99.376(2)°, *V=*1804.98(15) Å^3^, *ρ*=1.601 g cm^−3^, multiscan absorption correction using SADABS2016^[50],^ *μ*=3.371 mm^−1^, *T*_min_, *T*_max_=0.5094, 0.8894, 2*Θ* range 4.642–57.786°, reflections measured 54 208, independent reflections 9456 [R(int)=0.0442], 365 parameters, R‐index [*I*≥2*σ*(*I*)] 0.0323, *wR*_2_ (all data) 0.0540, GoF 1.034, Δ*ρ*_max_, Δ*ρ*_min_ 1.21/−0.80 e Å^3^.

**Crystal data of 6⋅1.5DCM**: C_29.5_H_51_Cl_3_I_2_O_8_Si_4_Sr, triclinic, *P*‐1, *Z*=4, 100(2) K, *a*=11.9953(8) Å, *b*=16.0514(12) Å, *c*=24.936(2) Å, *α*=76.567(6)°, *β*=85.494(6)°, *γ*=86.477(6)°, *V=*4650.7(6) Å^3^, *ρ*=1.562 g cm^−3^, numerical absorption correction using STOE X‐AREA and X‐RED32,^48^ *μ*=2.801 mm^−1^, *T*_min_, *T*_max_=0.5094, 0.8894, 2*Θ* range 4.642–57.786°, reflections measured 52 818, refined as a two component twin, 901 parameters, R‐index [*I*≥2*σ*(*I*)] 0.0780, *wR*_2_ (all data) 0.2325, GoF 0.871, Δ*ρ*_max_, Δ*ρ*_min_ 2.32/−1.74 e Å^3^.

## Conflict of interest

The authors declare no conflict of interest.
